# Serum neutrophil gelatinase-associated lipocalin as a potential signature for acute generalized exanthematous pustulosis and generalized pustular psoriasis: A case-control study

**DOI:** 10.1016/j.jdin.2025.10.002

**Published:** 2025-10-17

**Authors:** Yanhong Shou, Meijie Zhang

**Affiliations:** Department of Dermatology, The Second Affiliated Hospital, Zhejiang University, School of Medicine, Hangzhou, China

**Keywords:** AGEP, biomarkers, differential diagnosis, GPP, NGAL

*To the Editor:* Generalized pustular psoriasis (GPP) is a group of chronic or recurrent inflammatory skin diseases, characterized by sudden widespread sterile pustules and erythema, often accompanied by fever, fatigue, and laboratory abnormalities such as elevated C-reactive protein (CRP).^1^ Acute generalized exanthematous pustulosis (AGEP) is a rapid-onset, drug-induced or infection-induced pustular rash with systemic symptoms that resolve rapidly.^2^ Although the exact mechanisms of GPP and AGEP remain unclear, interleukin 1 and interleukin 36 are thought to drive keratinocyte-mediated inflammatory responses that promote neutrophil recruitment through chemokine release. Due to their similarities, distinguishing GPP from AGEP remains challenging.

While histopathological features showed substantial overlap, GPP was distinguished by subcorneal pustules with >50 neutrophils/hpf, contrasting with the predominant eosinophilic infiltrates in AGEP.^3^ Given the markedly neutrophil-dominant histopathology of GPP, we hypothesized that biomarkers associated with neutrophil activation or degranulation may help to differentiate these conditions. Neutrophil gelatinase-associated lipocalin (NGAL) can be detected early in acute kidney injury, which serves as a sensitive molecular indicator of neutrophil activation.^4^

We retrospectively analyzed clinical and laboratory data from 36 GPP patients and 11 AGEP patients diagnosed between 2019 and 2025 at the Department of Dermatology, the Second Affiliated Hospital, Zhejiang University. Cases with ambiguous clinical features were excluded. To fully explore the dermatologic relevance of NGAL in a real-world context, patients with renal dysfunction or comorbidities potentially affecting NGAL levels were not excluded (Supplementary Table I, available via Mendeley at https://data.mendeley.com/datasets/6y9kdn35yp/1). Baseline characteristics, including age, sex, white blood cell count, neutrophil count, CRP, erythrocyte sedimentation rate, creatinine, estimated glomerular filtration rate, and NGAL level, were comparable between groups (Table I). Serum NGAL levels were statistically higher in GPP patients than in AGEP patients (median: 285 vs 163 ng/mL, *P* = .009; Fig 1, *A*). To determine whether NGAL independently differentiates GPP from AGEP, we conducted a multivariable Firth logistic regression analysis incorporating NGAL alongside white blood cell, neutrophil count, CRP, erythrocyte sedimentation rate, creatinine, and estimated glomerular filtration rate. NGAL remained a significant independent predictor of GPP (*P* = .013; Supplementary Table II, available via Mendeley at https://data.mendeley.com/datasets/6y9kdn35yp/1). To assess the discriminative ability of serum NGAL between GPP and AGEP, we performed receiver operating characteristic curve analysis. NGAL demonstrated an area under the curve of 0.7588. At a threshold of 261 ng/mL, NGAL showed a Youden index of 0.583 (Fig 1, *B*). These results supported the potential utility of NGAL as a diagnostic marker, although further validation in larger cohorts was warranted.

**Table I:** Baseline characteristics of patients with AGEP and GPP

Parameters	AGEP	GPP
No.	11	36
Age, median (IQR)	40 (29.5, 60.5)	46 (31.5, 56)
Sex		
Female	5	15
Male	6	21
NGAL (ng/ml), median (IQR)	163 (78.5, 201)	285 (129.5, 445.75)
WBC (10ˆ9/L), median (IQR)	12.5 (9.65, 15.2)	10.2 (7.45, 14.48)
NE (10ˆ9/L), median (IQR)	9.7 (6.73, 11.52)	8.08 (5.76, 10.06)
CRP (mg/L), median (IQR)	32.80 (20.7, 59.6)	69.45 (16.2, 119.58)
ESR (mm/h), median (IQR)	15 (9.5, 19.5)	20 (7.75, 34.25)
Cr (μmol/L), median (IQR)	70.6 (59.5, 79.65)	65.5 (51.38, 76.75)
eGFR (mL/min), median (IQR)	109.69 (100.5, 117.99)	110.38 (94.78, 116.88)

*AGEP*, Acute generalized exanthematous pustulosis; *Cr*, creatinine; *CRP*, C-reactive protein; *eGFR*, estimated glomerular filtration rate; *ESR*, erythrocyte sedimentation rate; *GPP*, generalized pustular psoriasis; *IQR*, interquartile range; *NE*, neutrophil count; *NGAL*, neutrophil gelatinase-associated lipocalin; *WBC*, white blood cell.

**Fig 1:**
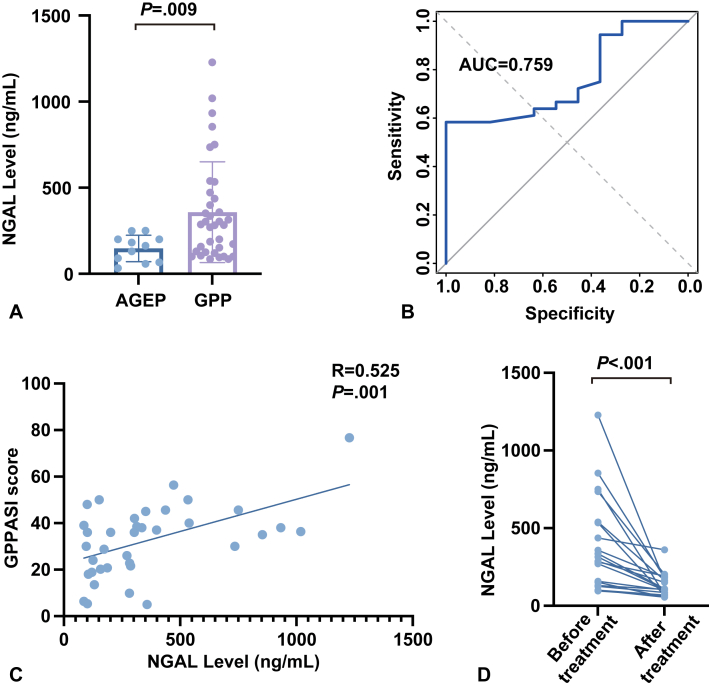
Differential levels of NGAL in GPP versus AGEP and its clinical implications. **A,** Serum NGAL levels in GPP and AGEP patients. Serum NGAL concentrations were statistically higher in patients with generalized pustular psoriasis (GPP, *n* = 36) compared to those with acute generalized exanthematous pustulosis (AGEP, *n* = 11) (Mann-Whitney *U* test, *P* = .009). **B,** Receiver operating characteristic (ROC) curve analysis of serum NGAL for discriminating GPP from AGEP. The area under the curve (AUC) was 0.7588. At a threshold of 261 ng/mL, NGAL showed a Youden index of 0.583. **C,** Correlation between serum NGAL and GPP disease severity. Serum NGAL levels statistically correlated with the GPPASI score (Spearman R = 0.525, *P* = .001). **D,** Dynamic changes in serum NGAL levels in GPP patients post-treatment. Following treatment, serum NGAL levels significantly decreased (Wilcoxon signed-rank test, *P* < .001). All data were represented as median (IQR). *AGEP*, Acute generalized exanthematous pustulosis; *GPP*, generalized pustular psoriasis; *GPPASI*, Generalized Pustular Psoriasis Area and Severity Index; *NGAL*, neutrophil gelatinase-associated lipocalin.

Notably, in GPP patients, serum NGAL levels positively correlated with disease severity, as measured by the Generalized Pustular Psoriasis Area and Severity Index (r = 0.525, *P* = .001; Fig 1, *C*). Among the 36 GPP patients, 20 (55.6%) underwent repeat NGAL testing immediately before discharge. Serum NGAL levels decreased significantly from a median of 300.5 ng/mL (interquartile range: 155-535.25) at admission to 105 ng/mL (interquartile range: 79.5-168.25) at discharge (*P* < .001, Wilcoxon signed-rank test; Fig 1, *D*), with the magnitude of reduction correlating with clinical improvement in pustule clearance.

Our study demonstrated that NGAL levels were significantly elevated in GPP correlating with the disease severity and declined with treatment. These observations may reflect involvement of NGAL in disease pathogenesis and activity monitoring, although the precise mechanisms remain unclear. However, NGAL levels could also partly reflect broader inflammatory processes. NGAL may serve as a supplementary biomarker to aid differentiation between GPP and AGEP, but should be interpreted alongside clinical and histopathological findings. NGAL testing has been incorporated into routine renal biomarker panels in many hospitals. The cost of an NGAL assay is approximately USD 6 per test, which is comparable to other conventional renal function tests, making it feasible for clinical practice. Nevertheless, our findings were based on a small, single-center cohort, and potential confounding factors cannot be fully excluded. Therefore, these observations are preliminary and warranted further validation in larger, multicenter studies to confirm their diagnostic and monitoring utility.

## Conflicts of interest

None disclosed.
